# Prediction of patient‐specific quality assurance for volumetric modulated arc therapy using radiomics‐based machine learning with dose distribution

**DOI:** 10.1002/acm2.14215

**Published:** 2023-11-21

**Authors:** Natsuki Ishizaka, Tomotaka Kinoshita, Madoka Sakai, Shunpei Tanabe, Hisashi Nakano, Satoshi Tanabe, Sae Nakamura, Kazuki Mayumi, Shinya Akamatsu, Takayuki Nishikata, Takeshi Takizawa, Takumi Yamada, Hironori Sakai, Motoki Kaidu, Ryuta Sasamoto, Hiroyuki Ishikawa, Satoru Utsunomiya

**Affiliations:** ^1^ Department of Radiology Niigata Prefectural Shibata Hospital Shibata City Niigata Japan; ^2^ Department of Radiological Technology Niigata University Graduate School of Health Sciences Niigata City Niigata Japan; ^3^ Department of Radiology Nagaoka Chuo General Hospital Nagaoka Niigata Japan; ^4^ Department of Radiation Oncology Niigata University Medical and Dental Hospital Niigata City Niigata Japan; ^5^ Department of Radiation Oncology Niigata Neurosurgical Hospital Niigata City Niigata Japan; ^6^ Department of Radiology Takeda General Hospital Aizuwakamatsu City Fukushima Japan; ^7^ Division of Radiology Nagaoka Red Cross Hospital Nagaoka‐shi Niigata Japan; ^8^ Section of Radiology, Department of Clinical Support Niigata University Medical and Dental Hospital Niigata City Niigata Japan; ^9^ Department of Radiology and Radiation Oncology Niigata University Graduate School of Medical and Dental Sciences Niigata City Niigata Japan

**Keywords:** machine learning, quality assurance, radiomics, volumetric modulated arc therapy

## Abstract

**Purpose:**

We sought to develop machine learning models to predict the results of patient‐specific quality assurance (QA) for volumetric modulated arc therapy (VMAT), which were represented by several dose‐evaluation metrics—including the gamma passing rates (GPRs)—and criteria based on the radiomic features of 3D dose distribution in a phantom.

**Methods:**

A total of 4,250 radiomic features of 3D dose distribution in a cylindrical dummy phantom for 140 arcs from 106 clinical VMAT plans were extracted. We obtained the following dose‐evaluation metrics: GPRs with global and local normalization, the dose difference (DD) in 1% and 2% passing rates (DD1% and DD2%) for 10% and 50% dose threshold, and the distance‐to‐agreement in 1‐mm and 2‐mm passing rates (DTA1 mm and DTA2 mm) for 0.5%/mm and 1.0%.mm dose gradient threshold determined by measurement using a diode array in patient‐specific QA. The machine learning regression models for predicting the values of the dose‐evaluation metrics using the radiomic features were developed based on the elastic net (EN) and extra trees (ET) models. The feature selection and tuning of hyperparameters were performed with nested cross‐validation in which four‐fold cross‐validation is used within the inner loop, and the performance of each model was evaluated in terms of the root mean square error (RMSE), the mean absolute error (MAE), and Spearman's rank correlation coefficient.

**Results:**

The RMSE and MAE for the developed machine learning models ranged from <1% to nearly <10% depending on the dose‐evaluation metric, the criteria, and dose and dose gradient thresholds used for both machine learning models. It was advantageous to focus on high dose region for predicating global GPR, DDs, and DTAs. For certain metrics and criteria, it was possible to create models applicable for patients’ heterogeneity by training only with dose distributions in phantom.

**Conclusions:**

The developed machine learning models showed high performance for predicting dose‐evaluation metrics especially for high dose region depending on the metric and criteria. Our results demonstrate that the radiomic features of dose distribution can be considered good indicators of the plan complexity and useful in predicting measured dose evaluation metrics.

## 1 INTRODUCTION

Volumetric modulated arc therapy (VMAT) is used frequently since it provides a conformal dose distribution to a target while achieving a dose reduction to organs at risk around the target. Although VMAT has clinical benefits including the quick delivery of treatment, it is considered a complex treatment technique due mainly to the complex motion of a multi‐leaf collimator (MLC) concurrent with gantry rotation; in addition, the dosimetric accuracy of VMAT for each treatment plan must be verified by patient‐specific quality assurance (QA) prior to treatment delivery.^1^ Gamma analysis is the most widely used method for comparisons between the measured dose distribution and the calculated dose distribution in patient‐specific QA for VMAT, with the degree of agreement usually quantified by using the gamma passing rate (GPR).^2^, ^3^ Dosimetric measurement as a part of patient‐specific QA is usually performed using multi‐dimensional detectors. Since patient‐specific QA is known to present a heavy workload, a much more efficient QA process is desirable, and obtaining such efficiency might be possible if the results of QA could be predicted accurately based on parameters involved in the created VMAT plan prior to the measurement.

There have been many investigations of the correlation between a plan‐complexity metric of VMAT plans and the GPRs.^4^, ^5^, ^6^, ^7^, ^8^, ^9^ The modulation complexity score for VMAT (MCSv) is a well‐known example that comprehensively quantifies the complexity of VMAT plans in terms of the MLC positions at each segment and the irregularity of the field shape.^5^ Although numerous complexity metrics have been introduced, only a few studies have indicated that a single complexity metric had a strong correlation with the GPR.^4^, ^7^ Machine learning‐based models in which multiple complexity metrics are used have been developed to accurately predict GPRs.^10^, ^11^, ^12^, ^13^, ^14^, ^15^, ^16^, ^17^, ^18^, ^19^, ^20^, ^21^, ^22^, ^23^ The machine learning models developed by Ono et al. showed high performance in predicting GPRs by combining multiple complexity metrics.^16^ More recently, a synthesized gamma map generated by a generative adversarial network was used to accurately predict failing points in the map and GPRs.^24^

Radiomic features of dose and fluence distributions were demonstrated to play important roles in the detection of errors and the prediction of GPRs in patient‐specific QA for intensity‐modulated radiation therapy (IMRT) and VMAT.^25^, ^26^, ^27^, ^28^, ^29^, ^30^, ^31^, ^32^, ^33^ For example, Park et al. showed that several textural features of two‐dimensional fluence maps of VMAT plans had a considerable correlation with the GPRs.^25^, ^26^, ^27^ Hirashima et al. proposed machine learning models that accurately predict the GPRs for VMAT plans by using the dosiomic features (radiomic features of dose distribution) incorporated in the conventional plan complexity metrics.^32^ Deep learning‐based GPR prediction models using coronal and sagittal 2D planar doses that showed a strong or moderate correlation between the measured and predicted GPRs were developed by Tomori et al., and interestingly, even though they used only “dummy target plans” that were created with a spherical phantom as the training dataset, the developed model also worked well for clinical target plans.^19^ These studies suggested that radiomic features of dose distributions alone could efficiently exhibit the complexity of each VMAT plan in the sense that GPRs could be accurately predicted by the features.

Although several research groups have developed machine learning models to predict GPRs based on radiomic features of dose distributions, several points have not been sufficiently addressed. The first point regards the dimension of dose distribution. Since VMAT produces a three‐dimensional (3D) modulation of the dose in a patient or a phantom by its nature, it is possible that the complexity of VMAT plans may be reflected more efficiently in a 3D dose distribution rather than a 2D dose distribution. Moreover, the 3D gamma analysis (which is an extension of the 2D gamma analysis into another dimension) may be more suitable for evaluating the entire volumetric dose distribution. A second point concerns the object at which the dose distribution is evaluated. Since the complexity of a VMAT plan is expected to reflect primarily the complexity of the beam delivery according to MLC motion and/or the irregularity of the field shape, it is reasonable to speculate that the VMAT plan's complexity has no direct relationship with the patient inhomogeneity correction.

At present, a typical VMAT patient‐specific QA procedure uses dose measurement by a phantom or multi‐dimensional detectors, and it may thus be reasonable to develop models to predict GPRs based on the dose distribution in a homogeneous phantom, which is expected to be more directly related to GPRs. In addition, it has been pointed out that radiomic features of dose distribution can vary according to the dose calculation algorithm and dose calculation grid size.^34^, ^35^, ^36^, ^37^ The use of a homogeneous phantom may help minimize such variation and obtain a robust prediction. A third point regards the selection of the normalization method and the criteria of dose evaluation metrics. The American Association of Physicists in Medicine (AAPM) Task Group (TG)−218 report stated that global normalization with the 3%/2 mm criterion is clinically relevant; however, one or more other metrics or criteria may be useful for predictions (e.g., local GPRs, passing rates based on the dose difference, or the distance‐to‐agreement alone), and there have been few systematic comparisons among multiple metrics and criteria.

In this study, we examined the predictive ability of several dose evaluation metrics and criteria based on the radiomic features of the 3D dose distribution of VMAT. We extracted the radiomic features from the dose distributions and created machine learning models based on those features to predict (i) 3D global and local GPRs, (ii) the passing rates based on the dose difference, and (iii) the distance‐to‐agreement alone determined by measurement with a multi‐dimensional detector array. We then evaluated the performance of each model. We used the so‐called “hand‐crafting” approach, in which the feature extraction and machine learning modeling are performed independently, as we speculated that doing so could enable us to observe a clear relationship between specific radiomic features and GPRs and the other dose evaluation metrics. We expected that, compared to deep learning‐based prediction, the hand‐crafting approach would make it much easier to interpret the results and compare them with the results of similar studies.^11^

## 2 MATERIALS AND METHODS

### 2.1 Workflow

**FIGURE 1:**
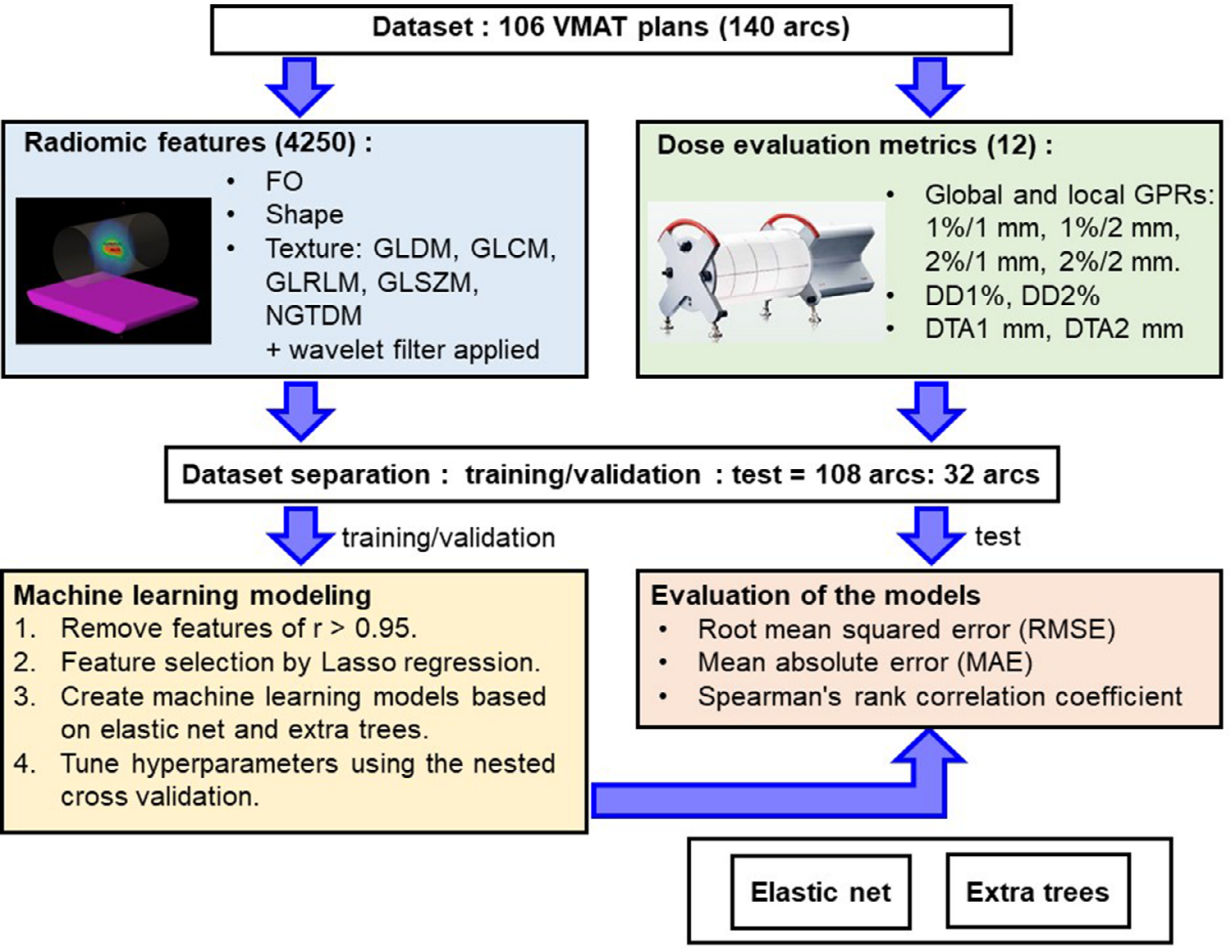
The study workflow. DDx%: passing rate for dose difference evaluation with x% criteria, DTAx mm: passing rate for distance‐to‐agreement in x mm criteria, FO: first order, GLCM: gray‐level co‐occurrence matrix, GLDM: gray‐level dependence matrix, GLRLM: gray‐level run‐length matrix, GLSZM: gray‐level size‐zone matrix, GPR: gamma passing rate, NGTDM: neighboring gray‐tone difference matrix.

As described in Figure 1, the overall workflow of our study was divided into the following five steps: (1) acquiring the dataset of clinical VMAT plans; (2) extracting radiomic features of 3D dose distributions in a dummy phantom calculated with the same arc setup as that used for clinical plans; (3) obtaining dose evaluation metrics such as GPRs by comparing the dose measured with the Delta4 multi‐dimensional volumetric dosimetry system (ScandiDos, Uppsala, Sweden) with the dose calculated using a treatment planning system (TPS); (4) separating the dataset into approximately 80% for training/validation and 20% for the test, and developing machine learning regression models to predict dose evaluation metrics with the use of the training/validation dataset based on the elastic net (EN) and the extra trees (ET); and (5) evaluating the performance of the developed models with the test dataset.

### 2.2 Datasets

We used the cases of 68 patients who underwent VMAT at our institute between October 2019 and March 2020. The numbers of patients for each treatment site (the total number of arcs) were 30 (30 arcs) for the prostate, 16 (51 arcs) for the head and neck, 18 (41 arcs) for the brain, 3 (12 arcs) for the whole pelvis, and 1 (6 arcs) for malignant pleural mesothelioma. The dose prescriptions of all of the treatments are summarized in Table 1. All of the clinical VMAT plans were created with the Eclipse treatment planning system ver. 15.5 (Varian Medical Systems, Palo Alto, CA). The anisotropic analytic algorithm (AAA, ver. 15.5) was used for the dose calculations, in which the dose grid size was 2.5 mm for the prostate, pelvis, and malignant pleural mesothelioma, 2.0 mm for the head and neck, and 1.25 mm for the brain. All of the clinical VMAT plans were delivered by a 6 MV photon beam and a Novalis Tx radiosurgery system (Varian Medical Systems) in which the MLC leaf width is 2.5 mm at the central area and 5 mm at the peripheral area. This retrospective study was approved by the institutional review board at our institute.

**TABLE 1:** Treatment sites, number of patients, total number of arcs, and prescription dose of the VMAT plans used.

Treatment site	No. of patients	No. of arcs	Dose prescriptions
Prostate	30	30	70 Gy in 28 fr. 39 Gy in 13 fr. (combined with HDR brachytherapy)
Head and neck	16	51	50 Gy in 25 fr. 60 Gy in 30 fr. 70 Gy in 35 fr.
Brain	18	41	18 Gy in 6 fr. 28 Gy in 4 fr. 60 Gy in 30 fr.
Whole pelvis	3	12	56 Gy in 28 fr. 76 Gy in 30 fr.
Malignant pleural mesothelioma	1	6	41.4 Gy in 23 fr. 12.6 Gy in 7 fr.

Abbreviations: fr.: fraction; HDR: high dose rate.

We obtained the GPRs with global and local normalization plus the dose difference (DD) of 1% (DD1%) and 2% (DD2%) and the distance‐to‐agreement (DTA) of the 1‐mm passing rate (DTA1 mm) and 2‐mm passing rate (DTA2 mm) by using the measured dose distributions with the Delta4 and the dose calculated with the TPS in a cylindrical, homogeneous numerical phantom (dia. 22 cm, length 40 cm; ScandiDos).^38^ The dose distribution in the phantom was calculated under the assumption that the phantom was irradiated by the clinical treatment beams with the dose calculation grid size of 2.0 mm. The computed tomography (CT) number of the phantom was set as 217 corresponding to the relative electron density of 1.147 in accordance with the specifications from ScandiDos.^38^ The 3D GPRs were obtained with a dose threshold of 10% for the following four criteria: 1%/1 mm, 1%/2 mm, 2%/1 mm, and 2%/2 mm. Criteria with larger dose differences, such as 3%/2 mm, were not included in this study, because their GPRs increased and could be concentrated around 100% when we performed the 3D gamma analysis.^39^, ^40^ We also evaluated the 3D passing rates for the DD1%, DD2% for a 10% dose threshold, DTA1 mm, and DTA2 mm for a 0.5%/mm dose gradient threshold. Additionally, the 3D GPRs, DD1%, and DD2% with the dose threshold of 50% threshold and DTA1 mm, and DTA2 mm for a 1.0%/mm dose gradient threshold were obtained and used to the additional machine learning modeling for comparison. All of the values of the dose evaluation metrics were calculated by the Delta4 software.^38^

### 2.3 Extraction of the radiomic features of the 3D dose distributions

A total of 106 radiomic features were extracted from the calculated 3D dose distributions in the Delta4 phantom with the use of 3D Slicer ver. 4.8.1 with PyRadiomics ver. 2.0.1.^41^, ^42^ The region of interest (ROI) used for the feature extraction was the region for the 10% or 50% dose threshold of the prescription dose. The 106 extracted features were classified into 18 first‐order features, 13 shape features, and 75 texture features. The texture features were classified into five classes: gray‐level dependence matrix (GLDM) (*n* = 14 features), gray‐level co‐occurrence matrix (GLCM) (*n* = 24), gray‐level run‐length matrix (GLRLM) (*n* = 16), gray‐level size‐zone matrix (GLSZM) (*n* = 16), and neighboring gray‐tone difference matrix (NGTDM) (*n* = 5). The details of the calculated radiomic features are provided in Table 2. All of the radiomic features were calculated for five different bin widths: 0.01, 0.1, 1.0, 10, and 100. We also obtained the features which 3D wavelet‐filter was applied at the first decomposition level consisting of 8 different blocks namely LLL, HLL, LHL, HHL, LLH, HLH, LHH, and HHH wavelet‐filters along the x‐, y‐, and z‐dimensions (where L = low‐pass filter and H = high‐pass filter). The effectiveness of wavelet‐filtered features in predicting gamma passing rate has been verified by Hirashima et al. in which the almost all selected useful radiomic features were wavelet‐filtered.^32^ Since the wavelet‐filter was not applied to the shape features, the total number of shape features was 65 (13 original features multiplied by 5 bin sizes), and the total number of the other features was 4185 (93 original features multiplied by 5 bin sizes and 9 patterns including 1 original and 8 wavelet‐filtered). The total number of features was thus 4,250.

**TABLE 2:** Number and class of the extracted radiomic features.

	Class	No. of features	Description
Statistical information	FO	18	Interquartile range, skewness, etc.
Shape‐based	Shape	13	Maximum 3D diameter, sphericity, etc.
Texture‐based	GLDM	14	Gray‐level variance, high gray‐level emphasis, etc.
	GLCM	24	Joint average, sum average, etc.
	GLRLM	16	Short‐run low gray‐level emphasis, gray‐level variance, etc.
	GLSZM	16	Gray‐level variance, zone variance, etc.
	NGTDM	5	Coarseness, complexity, etc.

Abbreviations: FO: first order; GLDM: gray‐level dependence matrix; GLCM: gray‐level co‐occurrence matrix; GLRLM: gray‐level run‐length matrix; GLSZM: gray‐level size‐zone matrix; NGTDM: neighboring gray‐tone difference matrix.

### 2.4 Machine learning modeling

Figure 1 is a schematic of the workflow of the development of the machine learning models. We created the machine learning models based on the EN and the ET by using the PyCaret program (ver. 2.3.10) with Python (ver. 3.8.16) to predict the global and local GPRs (1%/1 mm, 1%/2 mm, 2%/1 mm, and 2%/2 mm), DD1%, and DD2% for the 10% dose threshold, and DTA1 mm and DTA2 mm for the 0.5%/mm dose gradient threshold, respectively. The EN model is a regression model combining the Lasso and Ridge regularizations. The ET model is similar to the random forest algorithm, but it uses the whole original samples and randomly selects cut points in order to split nodes. The dataset was divided into 54 patients including 108 arcs for training/validation and other 14 patients including 32 arcs for testing, so that plans from a same patient were not divided into both the training and test dataset to avoid an information leakage.

Training and validation process described below were performed only with the ‘training/validation’ dataset and the ‘test’ dataset remained completely unknown for the created models. To avoid multi‐collinearity, the number of features was reduced by removing the features for which the correlation coefficients were > 0.95 (Figure 1). The radiomic features with a non‐zero coefficient were then selected using the Lasso regression using the PyCaret and created a ranking list of features. We created model based on the features from top of the list and tuned the hyperparameters of the EN and ET models by searching the optimal number of features that produces the minimum root mean squared error (RMSE) in a nested cross‐validation in which the four‐folds CV is used within the outer and inner loop. The performance of the developed models for predicting dose evaluation metrics with various criteria for the validation and test datasets was evaluated based on the RMSE, the mean absolute error (MAE), and Spearman's rank correlation coefficient (r).

The machine learning modeling processes mentioned above were repeated for dataset of the global and local GPRs (1%/1 mm, 1%/2 mm, 2%/1 mm, and 2%/2 mm), DD1%, and DD2% for the 50% dose threshold, and DTA1 mm and DTA2 mm for the 1.0%/mm dose gradient threshold. Finally, all the created models were applied to the test dataset of 3D dose distributions in patients’ CT images with heterogeneity and evaluated them in predicting dose evaluation metrics.

## 3 RESULTS

### 3.1 Dose evaluation metrics

Table 3 presents the measured values of the range, mean, standard deviation (SD_meas_), and median of the global and local GPRs with the criteria of 1%/1 mm, 1%/2 mm, 2%/1 mm, and 2%/2 mm and the DD1%, DD2% passing rates for the 10% and 50%, and DTA 1 mm, and DTA2 mm passing rates for the 0.5%/mm and 1.0%/mm dose gradient threshold, respectively. For more stringent criteria, the smaller mean and median values of the GPRs and larger range and SD_meas_ for the global and local GPRs were observed. The mean and median values of the global GPRs were always larger than those of the local GPRs for the same gamma analysis criterion. For all the criteria except the local 2%/1 mm and 2%/2 mm, SD_meas_ were larger for 50% threshold. The mean and median passing rates were the lowest and the range and SD_meas_ were the largest for DD1% among all of the dose evaluation metrics.

**TABLE 3:** The measured range, mean, SD_meas_, and median of the measured global and local GPRs, DDs passing rates with the dose threshold of 10% and 50%, and DTAs passing rates with the dose gradient threshold of 0.5%/mm and 1.0%/mm.

	Range [%]	Mean [%]	SD_meas_ [%]	Median [%]
10% dose threshold, 0.5% dose gradient threshold				
Global GPR 1%/1 mm	65.0−98.6	85.8	6.5	86.4
Global GPR 1%/2 mm	77.2−100.0	94.5	4.4	95.8
Global GPR 2%/1 mm	85.3−100.0	96.4	2.8	97.4
Global GPR 2%/2 mm	93.7−100.0	98.7	1.4	99.2
Local GPR 1%/1 mm	49.7−96.1	75.6	8.8	76.0
Local GPR 1%/2 mm	72.2−100.0	91.8	5.1	86.5
Local GPR 2%/1 mm	57.1−98.2	84.6	7.9	86.5
Local GPR 2%/2 mm	78.7−100.0	95.3	3.6	96.0
DD1%	39.9−81.0	63.8	9.1	65.3
DD2%	59.5−98.5	89.2	6.5	90.6
DTA1 mm	71.3−99.1	88.3	6.6	89.5
DTA2 mm	91.5−100.0	99.1	1.1	99.4
50% dose threshold, 1.0% dose gradient threshold				
Global GPR 1%/1 mm	48.2‐100.0	79.4	9.6	81.7
Global GPR 1%/2 mm	61.3‐100.0	91.4	7.4	94.1
Global GPR 2%/1 mm	70.0‐100.0	93.4	5.7	95.6
Global GPR 2%/2 mm	84.6‐100.0	97.4	3.0	98.6
Local GPR 1%/1 mm	40.8‐100.0	74.5	10.0	75.3
Local GPR 1%/2 mm	55.8‐100.0	89.9	7.8	92.1
Local GPR 2%/1 mm	66.5‐100.0	88.0	7.1	89.4
Local GPR 2%/2 mm	78.7‐100.0	95.7	3.8	96.8
DD1%	10.5‐80.1	51.6	14.7	53.0
DD2%	26.3‐97.5	81.5	13.6	85.2
DTA1 mm	59.4‐97.9	80.5	8.5	82.0
DTA2 mm	86.7‐100.0	95.8	2.8	96.2

Abbreviations: DDx%: passing rate for dose difference evaluation with the x% criterion; DTAx mm: passing rate for distance‐to‐agreement evaluation with the x mm criterion; GPR: gamma passing rate; SD: standard deviation; SD_meas_: standard deviation of measured dose evaluation metrics.

### 3.2 Prediction of dose evaluation metrics by the machine learning models

**FIGURE 2:**
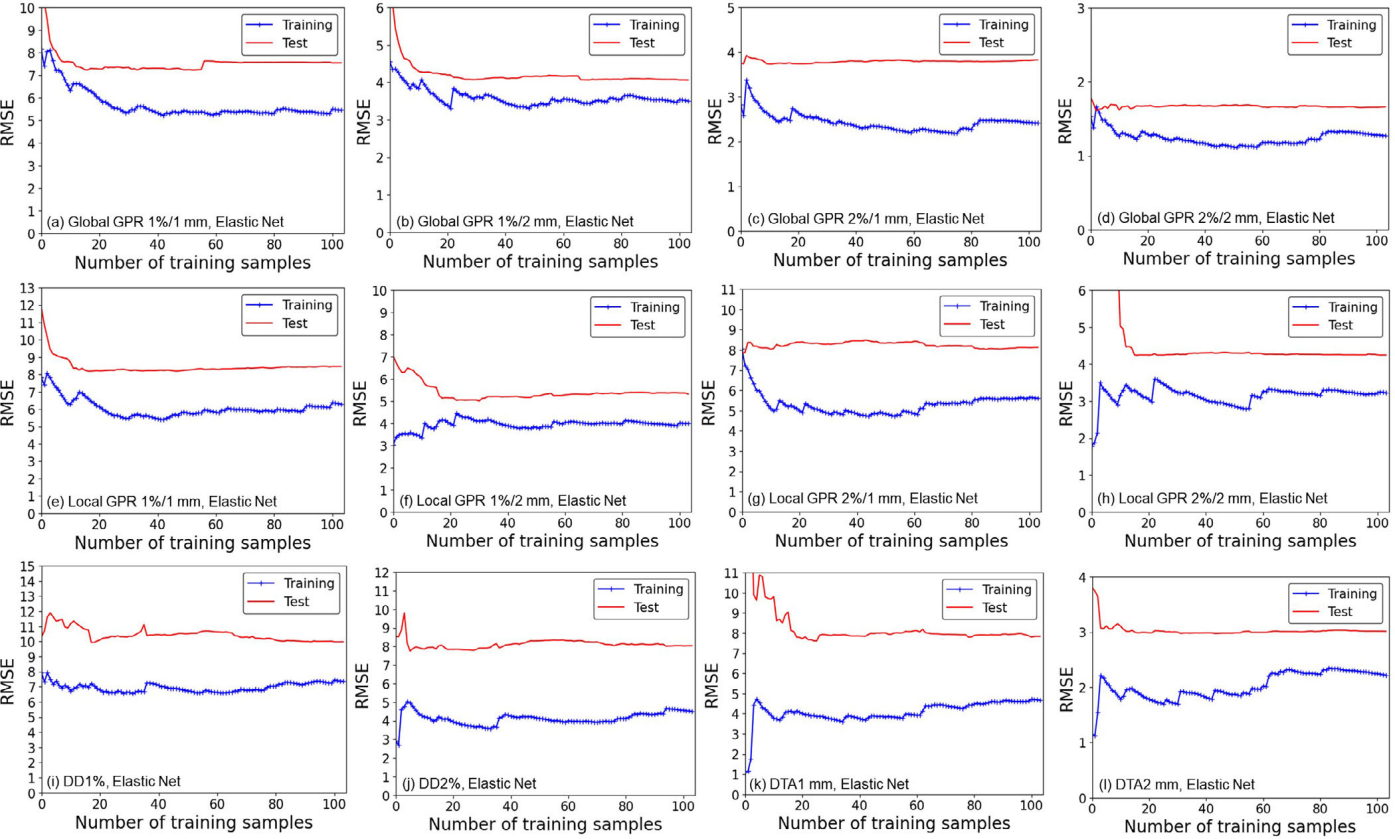
Learning curves of the elastic net (EN) models for global 1%/1 mm, 1%/2 mm, 2%/1 mm, and 2%/2 mm GPRs (*upper row*, a–d), local 1%/1 mm, 1%/2 mm, 2%/1 mm, and 2%/2 mm GPRs (*middle row*, e–h), and DD1%, DD2%, DTA1 mm, and DTA2 mm (*lower row*, i–l). Horizontal axes: the number of training samples. Vertical axes: the RMSE. *Blue lines*: training dataset. *Red lines*: test dataset. Abbreviations are explained in the Figure 1 legend. The dose threshold is 10% for GPRs, DD1%, and DD2%, and the dose gradient threshold is 0.5%/mm for DTA1 mm and DTA2 mm.

**FIGURE 3:**
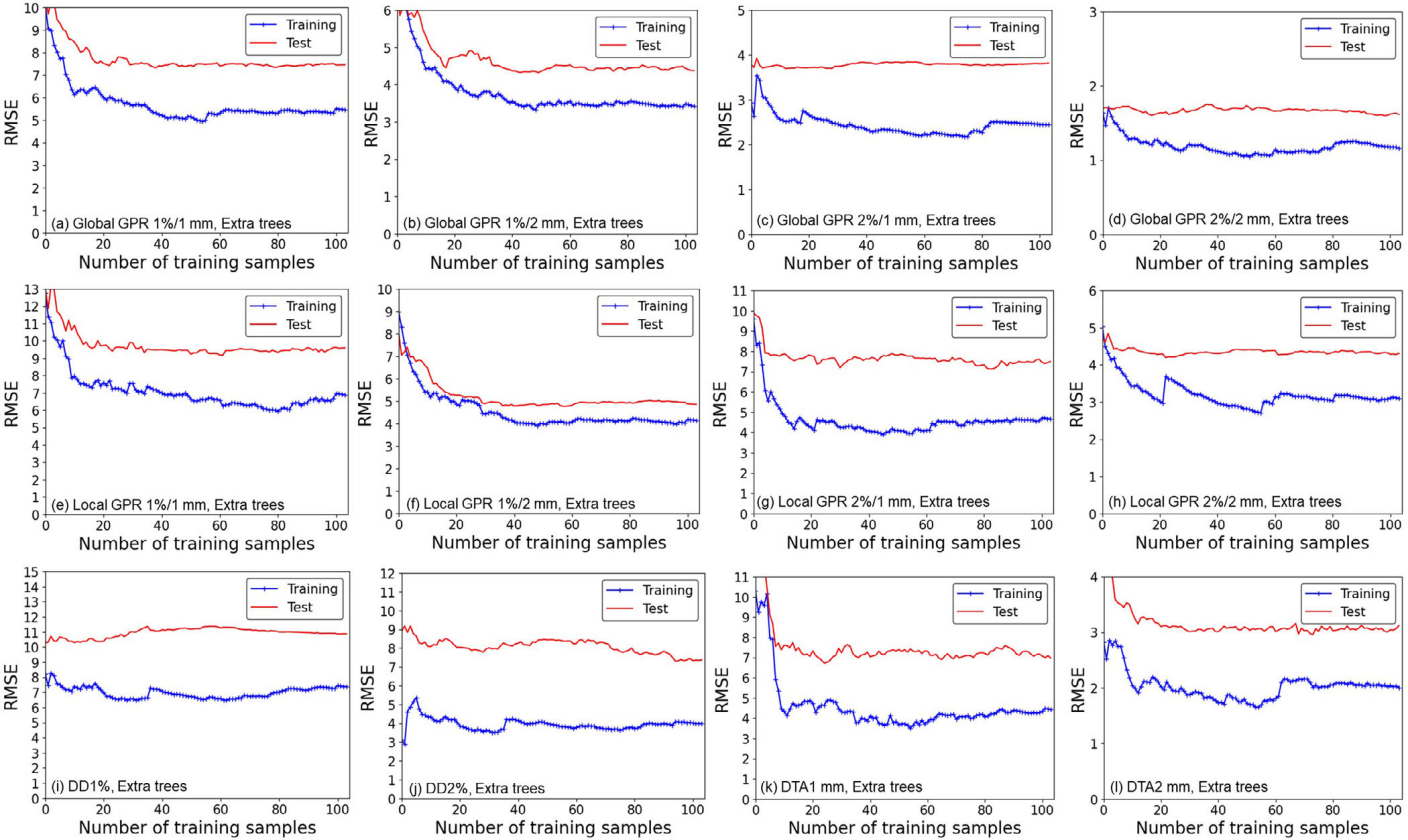
Learning curves of the extra trees (ET) models for global 1%/1 mm, 1%/2 mm, 2%/1 mm, and 2%/2 mm GPRs (*upper row*, a–d), local 1%/1 mm, 1%/2 mm, 2%/1 mm, and 2%/2 mm GPRs (*middle row*, e–h), and DD1%, DD2%, DTA1 mm, and DTA2 mm (*lower* row, i–l). Horizontal axes: the number of training samples. Vertical axes: the RMSE. *Blue lines*: training dataset. *Red lines*: test dataset. Abbreviations are explained in the Figure 1 legend. The dose threshold is 10% for GPRs, DD1%, and DD2%, and the dose gradient threshold is 0.5%/mm for DTA1 mm and DTA2 mm.

The learning curves in which the vertical axis represents the RMSE of the EN and ET models for the global and local GPRs, DD1%, DD2%, DTA1 mm, and DTA2 mm passing rates are shown in Figures 2 and 3, respectively. The training errors and the test errors became almost stable up to 60‐80 samples for all of the metrics and criteria. The shape of the training and test curves of the EN and ET were similar for all the metrics.

Table 4 presents the mean value and SDs of the predicted passing rates (SD_pred_) and the RMSEs, MAEs, and r‐values for the developed machine learning models based on the EN and ET for all of the criteria of dose evaluation metrics for 10% dose threshold (GPRs and DDs) and 0.5%/mm dose gradients threshold (DTAs). For the global GPRs with 10% dose threshold, the largest values of the RMSE and MAE for the training/validation dataset were 5.45% (EN) and 5.50% (ET) and 4.24% (EN) and 4.22% (ET) for the 1%/1 mm criterion, respectively. The largest values of the RMSE and MAE for local GPR values were also identified for the 1%/1 mm criterion, and the values were larger than those for the global GPRs. As the criteria became more stringent and the SD_pred_ became larger, the RMSE and MAE values became larger. The RMSE and MAE for test datasets were larger than training dataset for the EN and ET models overall. The highest correlation coefficients for training/validation were 0.81 (ET) for the DTA1 mm. The RMSE and MAE values for the training/validation of DD1% were 7.33% (EN) and 7.35% (ET) and 6.07% (EN) and 6.10% (ET), respectively, which were the largest values among all of the dose evaluation metrics, and the corresponding values for DTA2 mm were the smallest.

**TABLE 4:** The number of selected radiomic features, the mean and SD of the predicted values of metrics, the RMSE, MAE, Spearman's correlation coefficient, and RMSE/SD_meas_, where SD_meas_ is the standard deviation of measured values of metrics for the validation and test datasets for the EN and ET models with the dose threshold of 10% (global and local GPRs and DDs) and the dose gradient threshold of 0.5%/mm (DTAs).

	No. of selected features		Mean [%]		SD_pred_ [%]		RMSE [%]		MAE [%]		r		RMSE/SD_meas_	
	EN	ET	EN	ET	EN	ET	EN	ET	EN	ET	EN	ET	EN	ET
Training														
Global 1%/1‐mm	3	6	85.81	85.84	2.68	1.32	5.45	5.50	4.24	4.22	0.44	0.62	0.84	0.85
Global 1%/2‐mm	3	4	94.56	94.67	2.02	1.88	3.50	3.40	2.64	2.55	0.60	0.62	0.79	0.77
Global 2%/1‐mm	2	3	96.60	96.71	0.53	0.18	2.41	2.44	1.88	1.92	0.24	0.27	0.86	0.87
Global 2%/2‐mm	2	5	98.80	98.83	0.49	0.46	1.27	1.16	1.03	0.92	0.43	0.62	0.91	0.83
Local 1%/1‐mm	4	3	75.52	75.80	4.73	2.56	6.33	6.89	4.97	5.25	0.63	0.63	0.72	0.78
Local 1%/2‐mm	8	1	91.79	91.88	2.30	1.97	3.99	4.14	3.02	3.18	0.63	0.57	0.78	0.81
Local 2%/1‐mm	3	7	84.81	84.77	4.39	4.57	5.71	4.69	4.44	3.64	0.58	0.76	0.72	0.59
Local 2%/2‐mm	2	1	95.42	95.49	1.65	1.53	2.89	2.78	2.17	2.09	0.44	0.57	0.80	0.77
DD1%	4	1	64.51	64.49	3.78	3.51	7.33	7.35	6.07	6.10	0.50	0.50	0.81	0.81
DD2%	2	2	89.90	90.37	3.27	3.47	4.49	3.97	3.48	2.86	0.64	0.70	0.69	0.61
DTA1 mm	5	5	88.16	88.16	4.47	4.48	4.43	4.14	3.64	3.44	0.76	0.81	0.67	0.63
DTA2 mm	7	7	99.19	99.25	0.31	0.17	0.90	0.93	0.70	0.72	0.41	0.40	0.82	0.84
Test														
Global 1%/1‐mm			85.81	85.89	2.81	0.90	7.55	7.48	6.12	6.08	0.28	0.18	1.16	1.15
Global 1%/2‐mm			94.56	94.91	2.15	1.85	4.06	4.41	2.99	3.38	0.45	0.29	0.92	1.00
Global 2%/1‐mm			96.60	96.76	0.54	0.12	3.83	3.82	3.01	3.00	0.01	0.15	1.37	1.37
Global 2%/2‐mm			98.80	98.72	0.42	0.44	1.66	1.61	1.34	1.28	0.22	0.25	1.19	1.15
Local 1%/1‐mm			75.52	75.66	4.92	2.51	8.47	9.61	6.85	7.89	0.60	0.43	0.96	1.09
Local 1%/2‐mm			91.79	91.96	2.18	1.84	5.32	4.84	4.17	3.91	0.45	0.65	1.04	0.95
Local 2%/1‐mm			84.81	84.83	4.40	3.95	8.14	7.46	6.43	6.01	0.51	0.63	1.03	0.94
Local 2%/2‐mm			95.42	95.43	1.65	1.46	3.86	3.88	2.76	2.83	0.43	0.39	1.07	1.08
DD1%			64.51	64.29	3.45	3.49	10.02	10.92	8.06	8.81	0.35	0.27	1.10	1.20
DD2%			89.90	89.64	3.27	4.01	8.04	7.40	5.83	5.48	0.57	0.62	1.24	1.14
DTA1 mm			88.16	88.20	4.28	4.38	5.61	5.56	4.16	4.19	0.55	0.55	0.85	0.84
DTA2 mm			99.19	99.25	0.30	0.15	1.54	1.56	0.89	0.86	0.25	0.24	1.40	1.42

Abbreviations: DDx%: passing rate for dose difference evaluation with the x% criterion; DTAx mm: passing rate for distance‐to‐agreement evaluation with the x mm criterion; GPR: gamma passing rate; r: Spearman's rank correlation coefficient; SD_meas_: standard deviation of measured dose evaluation metrics; SD_pred_: standard deviation of predicted dose evaluation metrics.

**FIGURE 4:**
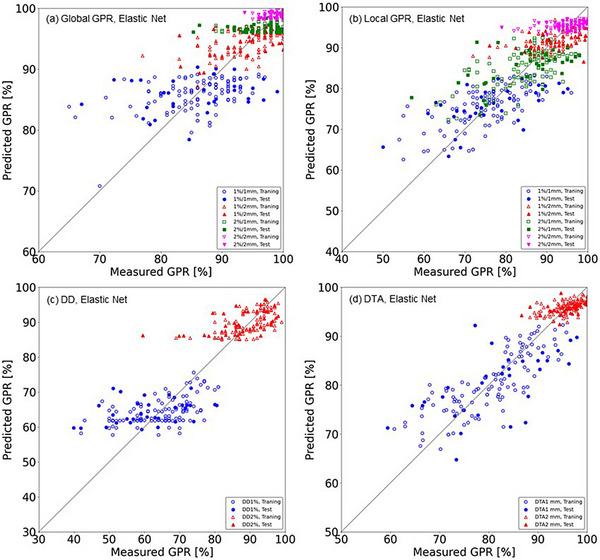
Scatterplots of the predicted and measured dose evaluation metrics in the elastic net (EN) model: (a) global GPRs for the 1%/1 mm, 1%/2 mm, 2%/1 mm, and 2%/2 mm criteria, (b) local GPRs for the 1%/1 mm, 1%/2 mm, 2%/1 mm, and 2%/2 mm criteria, (c) DD1% and DD2% criteria, and (d) DTA1 mm and DTA2 mm criteria. The dose threshold is 10% for GPRs, DD1%, and DD2%, and the dose gradient threshold is 0.5%/mm for DTA1 mm and DTA2 mm. *Diagonal line*: perfect prediction. Abbreviations are explained in the Figure 1 legend.

**FIGURE 5:**
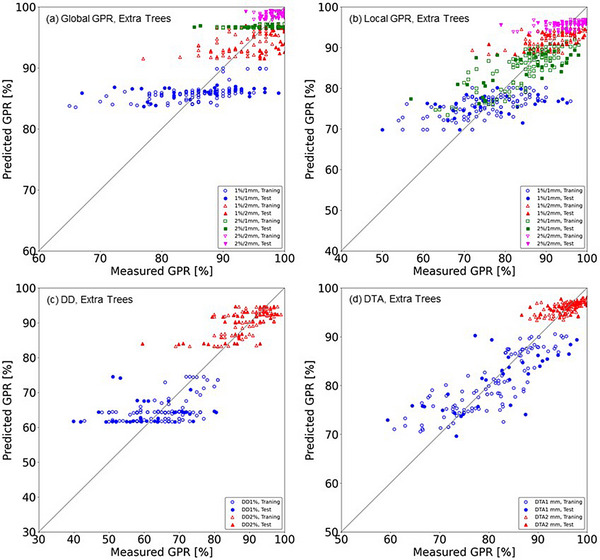
Scatterplots of the predicted and measured dose evaluation metrics in the extra trees (ET) model: (a) global GPRs for the 1%/1 mm, 1%/2 mm, 2%/1 mm, and 2%/2 mm criteria, (b) local GPRs for the 1%/1 mm, 1%/2 mm, 2%/1 mm, and 2%/2 mm criteria, (c) DD1% and DD2% criteria, and (d) DTA1 mm and DTA2 mm criteria. The dose threshold is 10% for GPRs, DD1%, and DD2%, and the dose gradient threshold is 0.5%/mm for DTA1 mm and DTA2 mm. *Diagonal line*: perfect prediction. Abbreviations are explained in the Figure 1 legend.

The RMSE divided by SD_meas_ (RMSE/SD_meas_)—where the SD_meas_ is the standard deviation of the measured global and local GPRs, DDs, and DTAs for training/validation and test datasets—were in the range of 0.59‐0.91 and 0.84‐1.42 for all of the dose evaluation metrics and machine learning models, respectively. It is observed that the values of RMSE/SD_meas_ were relatively small for the local GPRs with test dataset compared to the other metrics. Scatterplots of the measured and predicted global GPRs, local GPRs, DDs, and DTAs of the training/validation and test datasets by the EN model are provided in Figure 4. The distribution of values centered around the diagonal line, indicating perfect prediction for all of the metrics. Figure 5 depicts the results for the ET models in the same manner. Figures 4 and 5 show that the scatterplots for global GPRs and DDs revealed a pattern of predicting only specific values which appeared as an alignment in the scatterplot especially for the ET models. On the other hand, the scatterplots for local GPRs and DTA1 mm distributed centering around the diagonal line. Thus, the results that the values of RMSE/SD_meas_ were relatively small for the local GPRs and DTA1 mm compared to the other metrics confirmed graphically by these scatterplots.

Table 5 shows the results for 50% dose threshold (GPRs and DDs) and 1.0%/mm dose gradients threshold (DTAs), respectively. Although the values of the SD_pred_, RMSE, and MAE were much larger than Table 4, the values of RMSE/SD_meas_ were reduced except local GPRs. Especially for the DDs and DTAs, the much smaller values of RMSE/SD_meas_ were presented. Using a high dose region was advantageous to predict global GPRs, DDs, and DTAs accurately.

**TABLE 5:** The number of selected radiomic features, the mean and SD of the predicted values of metrics, DD1%, DD2%, DTA1 mm, and DTA2 mm, the MSE, MAE, Spearman's correlation coefficient, and RMSE/SD_meas_, where SD_meas_ is the standard deviation of measured values of metrics for the validation and test datasets for the EN and ET models with the dose threshold of 50% (global and local GPRs and DDs) and the dose gradient threshold of 1.0%/mm (DTAs).

	No. of selected features		Mean [%]		SD_pred_ [%]		RMSE [%]		MAE [%]		r		RMSE/SD_meas_	
	EN	ET	EN	ET	EN	ET	EN	ET	EN	ET	EN	ET	EN	ET
Training														
Global 1%/1‐mm	3	2	79.64	79.59	4.16	3.00	7.77	7.69	5.80	5.92	0.54	0.62	0.81	0.80
Global 1%/2‐mm	2	2	91.42	91.42	4.83	3.78	5.27	5.12	3.77	3.81	0.71	0.79	0.71	0.69
Global 2%/1‐mm	3	7	93.97	94.14	2.26	2.30	4.11	3.64	2.98	2.53	0.53	0.69	0.72	0.64
Global 2%/2‐mm	2	5	97.57	98.08	1.01	1.14	2.58	2.41	1.87	1.52	0.52	0.66	0.85	0.79
Local 1%/1‐mm	1	1	74.48	75.43	3.97	3.74	8.62	8.25	6.77	6.28	0.45	0.51	0.86	0.83
Local 1%/2‐mm	5	7	89.80	89.80	4.84	4.60	5.66	5.32	4.14	3.92	0.68	0.78	0.72	0.68
Local 2%/1‐mm	2	7	88.39	89.25	0.02	1.22	6.59	6.21	5.00	4.42	0.32	0.51	0.93	0.87
Local 2%/2‐mm	5	4	95.83	96.47	1.03	1.51	3.55	3.16	2.65	2.08	0.45	0.67	0.92	0.82
DD1%	9	4	53.12	53.93	10.64	11.32	8.71	8.23	6.95	6.52	0.76	0.81	0.59	0.56
DD2%	2	7	83.11	83.68	9.94	9.46	6.25	5.83	4.78	4.16	0.74	0.79	0.46	0.43
DTA1 mm	8	10	80.31	80.21	6.26	5.86	4.72	4.47	3.74	3.39	0.82	0.86	0.55	0.52
DTA2 mm	2	4	95.83	96.01	1.29	1.18	2.22	2.03	1.71	1.52	0.55	0.68	0.79	0.72
Test														
Global 1%/1‐mm			79.64	79.45	3.62	2.65	10.49	10.14	8.76	8.48	0.21	0.32	1.09	1.05
Global 1%/2‐mm			91.42	91.72	4.41	2.88	6.27	6.09	4.71	4.78	0.37	0.39	0.84	0.82
Global 2%/1‐mm			93.97	94.21	2.22	2.53	7.47	7.64	5.64	5.83	0.22	0.19	1.31	1.34
Global 2%/2‐mm			97.57	98.11	0.97	1.06	3.33	3.54	2.33	2.45	0.28	0.21	1.09	1.16
Local 1%/1‐mm			74.48	76.18	3.97	3.17	10.64	10.90	9.04	9.11	0.16	0.10	1.07	1.09
Local 1%/2‐mm			89.80	90.30	4.10	3.42	7.11	6.45	5.52	5.18	0.21	0.32	0.91	0.82
Local 2%/1‐mm			88.39	89.33	0.02	1.20	8.69	8.90	6.72	6.81	0.04	0.00	1.22	1.25
Local 2%/2‐mm			95.83	96.50	0.81	1.01	3.84	3.95	2.69	2.78	0.25	0.34	1.00	1.03
DD1%			53.12	54.18	9.36	10.83	13.98	14.52	11.57	12.22	0.53	0.57	0.95	0.99
DD2%			83.11	82.80	9.61	9.41	14.07	14.37	9.88	10.21	0.59	0.55	1.03	1.05
DTA1 mm			80.31	80.87	6.52	5.67	7.78	6.96	6.42	5.67	0.63	0.69	0.91	0.82
DTA2 mm			95.83	95.72	1.21	0.80	3.01	3.68	2.45	3.54	0.60	0.38	1.07	1.30

Abbreviations: DDx%: passing rate for dose difference evaluation with the x% criterion; DTAx mm: passing rate for distance‐to‐agreement evaluation with the x mm criterion; GPR: gamma passing rate; r: Spearman's rank correlation coefficient; SD_meas_: standard deviation of measured dose evaluation metrics; SD_pred_: standard deviation of predicted dose evaluation metrics.

Table 6 shows the results for that the created models were tested with the dataset which originated from dose distribution in patients’ CT images with heterogeneity for the dose threshold of 10% and 50% (GPRs and DDs), and 0.5%/mm and 1.0%/mm dose gradients threshold (DTAs). The RMSE, MAE, and RMSE/SD_meas_ increased and the r reduced for the same metric and criterion compared to the models based on the phantom overall. The exceptional cases were global 1%/1 mm, global 2%/2 mm, and local 2%/2 mm GPR with 50% dose threshold for which the created models maintained a small RMSE/SD_meas_ and a high r even with test dataset with patients’ heterogeneity.

**TABLE 6:** The mean and SD of the predicted values of metrics, DD1%, DD2%, DTA1 mm, and DTA2 mm, the MSE, MAE, Spearman's correlation coefficient, and RMSE/SD_meas_, where SD_meas_ is the standard deviation of measured values of metrics for the validation and test datasets for the EN and ET models for that the test data set which originated from dose distribution in patient with heterogeneity with the dose threshold of 10% and 50% (global and local GPRs and DDs), and dose gradient threshold of 0.5%/mm and 1.0%/mm (DTAs).

	Mean [%]		SD_pred_ [%]		RMSE [%]		MAE [%]		r		RMSE/SD_meas_	
	EN	ET	EN	ET	EN	ET	EN	ET	EN	ET	EN	ET
10% dose threshold, 0.5%/mm dose gradient threshold												
Global 1%/1‐mm	85.81	85.92	2.37	0.92	8.03	7.61	6.60	6.30	0.19	0.22	1.23	1.17
Global 1%/2‐mm	94.56	94.98	1.70	1.34	5.63	5.06	4.40	3.83	‐0.23	0.12	1.28	1.15
Global 2%/1‐mm	96.60	96.75	0.49	0.15	3.87	3.85	3.12	3.05	‐0.01	‐0.04	1.38	1.38
Global 2%/2‐mm	98.80	98.82	0.45	0.32	1.58	1.61	1.22	1.24	0.37	0.36	1.13	1.15
Local 1%/1‐mm	75.52	75.76	4.96	2.29	11.75	10.76	9.00	8.66	0.05	0.03	1.34	1.22
Local 1%/2‐mm	91.79	92.00	1.89	1.84	5.84	6.04	4.66	4.80	0.15	0.07	1.14	1.18
Local 2%/1‐mm	84.81	84.91	4.41	2.98	10.36	10.07	8.01	8.03	0.12	0.11	1.31	1.28
Local 2%/2‐mm	95.42	95.51	0.84	0.83	4.44	4.35	3.23	3.11	0.03	0.29	1.23	1.21
DD1%	64.51	65.06	2.31	2.45	11.43	11.42	9.43	9.33	‐0.30	0.09	1.26	1.26
DD2%	89.90	91.25	3.27	1.57	9.50	9.77	7.52	7.53	‐0.21	0.02	1.46	1.50
DTA1 mm	88.16	88.39	3.33	3.29	7.41	7.03	6.04	5.71	0.02	0.12	1.12	1.07
DTA2 mm	99.19	99.28	0.30	0.12	1.64	1.62	0.90	0.87	0.11	0.02	1.49	1.48
50% dose threshold, 1.0%/mm dose gradient threshold												
Global 1%/1‐mm	79.64	79.44	2.56	1.90	10.51	10.11	8.56	8.30	0.23	0.42	1.09	1.05
Global 1%/2‐mm	91.42	91.40	4.39	2.58	7.64	8.20	6.47	6.74	0.18	‐0.09	1.03	1.10
Global 2%/1‐mm	93.97	93.87	1.61	1.95	7.85	8.22	5.63	6.00	0.17	‐0.02	1.38	1.44
Global 2%/2‐mm	97.57	97.75	0.76	0.90	3.25	3.38	2.39	2.36	0.51	0.25	1.07	1.11
Local 1%/1‐mm	74.48	75.89	3.97	3.50	11.26	11.18	9.89	9.92	0.17	0.13	1.13	1.12
Local 1%/2‐mm	89.80	90.21	3.33	2.06	7.60	8.27	6.27	6.77	0.26	‐0.12	0.97	1.05
Local 2%/1‐mm	88.39	89.46	0.01	1.04	8.69	9.02	6.72	6.82	0.22	0.06	1.22	1.27
Local 2%/2‐mm	95.83	96.34	0.48	1.04	3.76	3.72	2.77	2.81	0.46	0.37	0.98	0.97
DD1%	53.12	55.48	7.74	8.21	18.13	15.72	15.10	12.54	‐0.08	0.46	1.24	1.07
DD2%	83.11	84.83	6.17	4.10	18.03	17.68	12.27	12.07	0.13	0.32	1.32	1.30
DTA1 mm	80.31	80.86	3.70	2.89	9.83	8.99	8.12	7.31	0.19	0.43	1.15	1.05
DTA2 mm	95.83	96.13	0.91	0.64	3.50	3.43	2.78	2.70	0.12	0.05	1.24	1.21

Abbreviations: DDx%: passing rate for dose difference evaluation with the x% criterion; DTAx mm: passing rate for distance‐to‐agreement evaluation with the x mm criterion; GPR: gamma passing rate; r: Spearman's rank correlation coefficient; SD_meas_: standard deviation of measured dose evaluation metrics; SD_pred_: standard deviation of predicted dose evaluation metrics.

The selected radiomic features in the machine learning modeling are summarized in Tables 7, 8, 9 for the global GPRs, local GPRs, and DDs and DTAs, respectively. The number of selected radiomic features ranged from 1 to 10 depending on the dose evaluation metric and criteria. The maximum number of features was 10 for the DTA1 mm with 1.0%/mm dose gradient threshold, and the second‐highest number was 9 for the DD1% with 50% dose threshold. There are some features which were present in multiple metrics and criteria. The 28 features were selected in multiple criteria. Especially, the “zone entropy” was selected for 11 criteria, the “gray‐level nonuniformity” was selected for 10 criteria, the “dependence variance” was selected for 9 criteria, and the “Imc1” was selected for 7 criteria. The class of the most majority was the GLSZM. There was no particular bin width that was selected more than the others. In addition, the great majority of the features listed in Tables 7, 8, 9 were features in which a wavelet‐filter was applied.

**TABLE 7:** Selected radiomic features in descending order of the absolute value of coefficients of Lasso regression with the dose threshold of 10% and 50% are displayed with the bin width, the class, and the wavelet‐filter for global GPRs.

1%/1 mm, 10% threshold					1%/2 mm, 10% threshold			
	Bin width	Class	Feature	WF	Bin width	Class	Feature	WF
1	0.01	GLSZM	Gray‐level nonuniformity	LLH	0.01	GLSZM	Size zone nonuniformity	LLH
2	100	GLCM	Imc2	LHH	0.01	GLRLM	Run entropy	HLH
3	0.01	GLCM	Idn	LLH	0.01	GLSZM	Gray‐level nonuniformity	LHH
4	0.01	GLSZM	Size zone nonuniformity normalized	HLH	0.01	GLRLM	Run length nonuniformity normalized	LLH
5	0.1	GLSZM	Zone variance	HLL				
6	0.1	GLCM	Imc1	LLL				

2%/1 mm, 10% threshold					2%/2 mm, 10% threshold			
	Bin width	Class	Feature	WF	Bin width	Class	Feature	WF
1	100	GLSZM	Zone variance	LHH	0.1	GLSZM	Zone variance	HLL
2	10	GLSZM	Zone variance	HLH	1.0	GLDM	Dependence variance	LLH
3	0.1	GLRLM	Long run emphasis	LLL	0.1	GLRLM	Run entropy	HHL
4					0.01	NGTDM	Complexity	LLH
5					1.0	FO	Kurtosis	HLH

1%/1 mm, 50% threshold					1%/2 mm, 50% threshold			
	Bin width	Class	Feature	WF	Bin width	Class	Feature	WF
1	0.01	GLSZM	Zone entropy	HLH	1.0	GLDM	Dependence variance	LLL
2	100	GLCM	Imc1	LLH	1.0	GLCM	Imc1	LLH
3	1.0	GLDM	Dependence variance	LLL				

2%/1 mm, 50% threshold					2%/2 mm, 50% threshold			
	Bin width	Class	Feature	WF	Bin width	Class	Feature	WF
1	0.01	GLDM	Dependence variance	HLL	100	GLCM	Imc1	LLH
2	1.0	GLRLM	Run length nonuniformity normalized	LLL	0.01	GLDM	Dependence entropy	HLH
3	0.01	GLCM	Idmn	LLH	1.0	FO	Range	HLH
4	1.0	GLRLM	Long run high gray‐level emphasis	LLH	1.0	GLSZM	Zone entropy	HLL
5	0.01	GLCM	Idn	LLH	0.01	GLCM	Idn	LLH
6	1.0	GLSZM	Zone entropy	LLH				
7	0.01	GLCM	Cluster shade	HLL				

*Note*: The number of selected features depending on the machine learning model are presented in Tabled 4, 5.

Abbreviations: FO: first order; GLCM: gray‐level co‐occurrence matrix; GLDM: gray‐level dependence matrix; GLRLM: gray‐level run‐length matrix; GLSZM: gray‐level size‐zone matrix; GPR: gamma passing rate; LLL/HLL/LHL/HHL/LLH/HLH/LHH/HHH: wavelet‐filters along x‐, y‐, and z‐dimensions applied (L and H: low‐pass and high‐pass filters, respectively); NGTDM: neighboring gray‐tone difference matrix; ORG: the original value of the feature with a wavelet‐filter not applied; WF: wavelet‐filter.

**TABLE 8:** Selected radiomic features in descending order of the absolute value of coefficients of Lasso regression with the dose threshold of 10% and 50% are displayed with the bin width, the class, and the wavelet‐filter for local GPRs.

1%/1 mm, 10% threshold					1%/2 mm, 10% threshold			
	Bin width	Class	Feature	WF	Bin width	Class	Feature	WF
1	0.01	GLRLM	Run length nonuniformity	LLH	0.01	GLSZM	Gray level nonuniformity	LLH
2	0.01	GLCM	Idm	HHL	10	GLDM	Large dependence high gray‐level emphasis	LHL
3	100	GLSZM	Gray level nonniformity	HHL	0.01	GLCM	Maximum probability	LLH
4	0.01	GLDM	Dependence nonuniformity	LLH	0.01	GLSZM	Gray level nonuniformity	LHH
5					0.1	FO	Kurtosis	HHL
6					1.0	GLCM	Idm	LLH
7					100	GLSZM	Zone entropy	HLL
8					1.0	GLCM	Imc2	LLH

2%/1 mm, 10% threshold					2%/2 mm, 10% threshold			
	Bin width	Class	Feature	WF	Bin width	Class	Feature	WF
1	10	GLRLM	Run variance	LLL	0.01	GLRLM	Run length nonuniformity	LLH
2	0.1	GLSZM	Large area high gray‐level emphasis	LLH	100	GLCM	Idm	LHH
3	1.0	GLRLM	Short run high gray‐level emphasis	LHH				
4	100	GLSZM	Zone entropy	HLL				
5	1.0	GLSZM	Size zone nonuniformity	HHL				
6	1.0	GLRLM	Run variance	ORG				
7	0.1	GLRLM	Gray‐level nonuniformity	LLL				

1%/1 mm, 50% threshold					1%/2 mm, 50% threshold			
	Bin width	Class	Feature	WF	Bin width	Class	Feature	WF
1	0.01	GLSZM	Gray Level nonuniformity	LHH	1.0	GLDM	Dependence variance	LLL
2					1.0	GLCM	Imc1	LLH
3					0.01	GLDM	Large dependence high gray‐level emphasis	HLH
4					1.0	FO	Range	HLH
5					0.1	GLDM	Dependence variance	ORG
6					0.1	GLCM	Joint average	HHL
7					0.1	GLDM	Small dependence emphasis	ORG

2%/1 mm, 50% threshold					2%/2 mm, 50% threshold			
	Bin width	Class	Feature	WF	Bin width	Class	Feature	WF
1	100	GLRLM	Short run high gray‐level emphasis	LHL	1.0	GLCM	Imc1	LLH
2	0.1	GLDM	Dependence variance	LLH	0.01	GLSZM	Gray‐level nonuniformity	LHH
3	1.0	GLSZM	Size zone nonuniformity normalized	LLL	0.01	GLDM	Dependence variance	LLH
4	1.0	GLRLM	Long run emphasis	LLH	0.1	GLSZM	Zone entropy	HHL
5	100	GLCM	Autocorrelation	LHL	0.01	GLDM	Large dependence high gray‐level emphasis	HLH
6	1.0	GLCM	Cluster prominence	LLH				
7	1.0	GLCM	Cluster shade	LLL				

*Note*: The number of selected features depending on the machine learning model are presented in Tables 4, 5.

Abbreviations: FO: first order; GLCM: gray‐level co‐occurrence matrix; GLDM: gray‐level dependence matrix; GLRLM: gray‐level run‐length matrix; GLSZM: gray‐level size‐zone matrix; GPR: gamma passing rate; LLL/HLL/LHL/HHL/LLH/HLH/LHH/HHH: wavelet‐filters along x‐, y‐, and z‐dimensions applied (L and H: low‐pass and high‐pass filters, respectively); NGTDM: neighboring gray‐tone difference matrix; ORG: the original value of the feature with a wavelet‐filter not applied; WF: wavelet‐filter.

**TABLE 9:** Selected radiomic features in descending order of the absolute value of coefficients of Lasso regression with the dose threshold of 10% and 50% (DDs) and the dose gradient threshold of 0.5%/mm and 1.0 %/mm (DTAs) are displayed with the bin width, the class, and the wavelet‐filter for DDs and DTAs.

DD1%, 10% threshold					DD2%, 10% threshold			
	Bin width	Class	Feature	WF	Bin width	Class	Feature	WF
1	0.01	GLSZM	Large area high gray‐level emphasis	LLH	0.1	NGTDM	Busyness	LHH
2	0.01	GLSZM	Zone entropy	HLH	1.0	FO	Mean absolute deviation	LLH
3	0.01	GLSZM	Zone entropy	LHL				
4	100	GLSZM	Zone entropy	LHH				

DTA1 mm, 0.5%/mm threshold					DTA2 mm, 0.5%/mm threshold			
	Bin width	Class	Feature	WF	Bin width	Class	Feature	WF
1	0.1	GLSZM	Gray level nonuniformity	LHL	0.01	GLSZM	Gray level nonuniformity	LLH
2	0.1	GLCM	Imc2	LLH	0.1	GLRLM	Run entropy	LLH
3	0.01	GLRLM	Run length nonuniformity	LLH	1.0	GLSZM	Small area low gray‐level emphasis	ORG
4	0.1	GLSZM	Size zone nonuniformity normalized	ORG	0.01	NGTDM	Contrast	HHH
5	100	GLSZM	High gray‐level zone emphasis	HLL				
6	0.1	GLSZM	Zone entropy	LHH				
7	1.0	GLCM	Idmn	LLH				
8	0.01	GLSZM	Size zone nonuniformity normalized	HHH				
9	0.1	GLSZM	Size zone nonuniformity normalized	LLL				
10	0.1	GLRLM	Long run emphasis	LLH				

DD1%, 50% threshold					DD2%, 50% threshold			
	Bin width	Class	Feature	WF	Bin width	Class	Feature	WF
1	100	GLDM	Dependence variance	HHL	0.01	GLDM	Dependence variance	HHH
2	0.01	GLDM	Dependence entropy	HLH	100	GLRLM	Run length nonuniformity normalized	HHH
3	10	GLSZM	Small area high gray‐level emphasis	HLH	10	GLSZM	Small area high gray‐level emphasis	HLH
4	0.01	GLCM	Idn	LLH	0.1	NGTDM	Busyness	HLL
5	100	GLDM	Dependence variance	LHL	0.1	GLDM	Small dependence low gray‐level emphasis	LHH
6	100	GLRLM	Short run low gray‐level emphasis	HHH	0.01	GLSZM	Size zone nonuniformity normalized	LLH
7	1.0	GLSZM	Small area low gray‐level emphasis	LHH	1.0	GLSZM	Zone entropy	LLH
8	0.1	GLCM	Imc1	HHL				
9	100	FO	Skewness	LHL				

DTA1 mm, 1.0%/mm threshold					DTA2 mm, 1.0%/mm threshold			
	Bin width	Class	Feature	WF	Bin width	Class	Feature	WF
1	10	GLRLM	Run variance	LLL	0.1	GLSZM	Zone entropy	LLL
2	0.1	GLRLM	Gray level nonuniformity	LLL	0.1	GLRLM	Long run emphasis	HLL
3	0.01	GLDM	Large dependence emphasis	LHH	1.0	NGTDM	Complexity	LLL
4	100	GLSZM	Zone entropy	HLL	0.1	NGTDM	Busyness	LLL
5	100	GLSZM	Gray‐level nonuniformity normalized	HLH	0.1	FO	Kurtosis	HHL
6					0.1	GLDM	Large dependence emphasis	HHL
7					100	GLDM	Large dependence emphasis	HHH

*Note*: The number of selected features depending on the machine learning model are presented in Table 4, 5.

Abbreviations: DDx%: passing rate for dose difference evaluation with the x% criterion; DTAx mm: passing rate for distance‐to‐agreement evaluation with the x mm criterion; FO: first order; GLCM: gray‐level co‐occurrence matrix; GLDM: gray‐level dependence matrix; GLRLM: gray‐level run‐length matrix; GLSZM: gray‐level size‐zone matrix, GPR: gamma passing rate; LLL/HLL/LHL/HHL/LLH/HLH/LHH/HHH: wavelet‐filters along x‐, y‐, and z‐dimensions applied (L and H: low‐pass and high‐pass filters, respectively); NGTDM: neighboring gray‐tone difference matrix; ORG: the original value of the feature with a wavelet‐filter not applied; WF: wavelet‐filter.

## 4 DISCUSSION

The performance of our developed machine learning models for predicting global and local GPRs, DDs, and DTAs are summarized in Table 4 and presented graphically in Figures 4 and 5 as scatterplots of the predicted and measured passing rates. The developed models showed accuracy at approx. 1%−10% of the RMSE for the test dataset in predicting the dose evaluation metrics and criteria overall. According to the learning curves shown in Figures 2 and 3 and the results in Table 4, although the EN and ET models showed the larger RMSE and MAE values for test datasets than the training dataset, it suggests that the models acquired a sufficient level of generalization performance if those are compared to the results of Hirashima et al in which the RMSE for test dataset was more than double of that for validation dataset.^31^ The EN and ET models showed comparable results overall implying that the obtained results are robust and mostly regardless to the selection of the machine learning algorithm. The RMSE and MAE values for the GPRs were higher for the more stringent criteria, that is, those with a smaller dose difference and DTA. This may be simply understood by noting that the SDs of the measured GPRs were larger for stringent criteria, as suggested by the data in Table 4. The ratio of the RMSE to the SD of the measured dose evaluation metric for the EN and ET models were in a narrow range, that is, 0.59‐0.91 (training) and 0.84‐1.42 (test), suggesting that the RMSE increases as the SD of the measured dose evaluation metric increases universally, and then the ratios are considered a good indicator of predicting accuracy and a single use of the RMSE or MAE may not be sufficient.

The results for 50% dose threshold (Table 5) showed a superior accuracy of prediction according to the ratio of the RMSE to the SD of the measured dose evaluation metric except local GPRs. A possible reason for this may be that dose distribution higher than 50% is more likely to reflects the characteristics of the treatment plan and more closely related to the complexity of the plan than the lower dose. Compared to the other metrics, the SD of the measured values of local GPRs were not changed very much for 10% and 50% dose threshold as shown in Table 3. The less variability of GPR may be the reason for that the prediction accuracy of local GPRs exhibited inferior for 50% dose threshold.

The poor accuracy of the created models based on dose distribution in the phantom in applying for test dataset originated from dose distribution in patients’ CT images with heterogeneity as shown in Table 6 suggested that there is a limitation of applying the models. It implied that only dataset originated from phantom may be not sufficient to accurately predict all of the dose evaluation metrics by dose distribution in patients’ CT images with heterogeneity. However, the global 1%/1 mm, global 2%/2 mm, and local 2%/2 mm GPR with 50% dose threshold considerably maintained a comparable prediction accuracy to the pure phantom study, exhibiting a high capability of the created model in applying them to a calculated dose distribution with patients’ heterogeneity.

The selected radiomic features were distributed among the first‐order features and the five texture features classes as shown in Tables 7, 8, 9. There was no prominently important features or class, and it was concluded that a combination of features was required to accurately predict dose evaluation metrics. It is notable that there was no shape feature listed in Tables 7, 8, 9 which include descriptors of the size and shape of the selected region of dose distribution. It implied that the size and shape of the selected region of dose distribution may not be an indicator of the complexity of the VMAT plan and irrelevant to the prediction dose evaluation metrics.

The performance of our developed models can be closely compared to the “dosiomics model” presented by Hirashima et al. and the deep learning‐based models presented by Tomori et al.^19^, ^31^ The RMSE for the global GPR of 2%/2 mm was 1.2% for our EN model; the value reported by Hirashima et al. for the same criterion was 5.7%. However, this superior accuracy may be due primarily to the difference in the SD of the measured value of the GPRs. The SD of the measured GPR of 2%/2 mm reported by Hirashima et al. was 7.4%, resulting in an RMSE to SD ratio of 0.77, which is close to the range of our results presented in Table 4. The reason that the SDs of our models were significantly smaller than those reported by Hirashima et al. may be that we used a 3D gamma evaluation, which produces higher and more compactly distributed passing rates than a 2D gamma evaluation.^38^, ^39^ Our models showed slightly superior accuracy compared to the models developed by Tomori et al. for the similar SD values of the measured global GPRs.^19^ This could also be attributed to their larger SDs of the measured GPRs, that is, 2.94% for the 2%/2 mm GPR. The RMSE to SD ratio for their results was 0.90. Collectively, these findings suggest that the ratio of the RMSE to the SD of measured GPRs is approximately constant regardless of the prediction model and the detector used for the GPR measurement. Therefore, the accuracy of prediction models must be evaluated by the conventional metrics (MAE and RMSE) in association with the variation (SD) of the measured objective.

Hirashima et al. reported that the class of GLDM was the most important parameter for predicting global GPRs.^31^ Two of the top‐ten ranked radiomic features in the Hirashima et al. study were also selected in our global GPR results, namely the “dependence entropy” and the “dependence variance.” These features are thus thought to represent universal characteristics of dose distribution that are relevant to the global GPRs. In this work, we adopted the so‐called hand‐crafting approach rather than a deep learning‐based approach. The step‐by‐step process of the hand‐crafting approach enabled us to specify the effective features for each metric and criterion and to determine what they have in common with other criteria. It also enabled us to compare the effective features with those described in similar studies and to identify universally important features of VMAT dose distribution. Moreover, since we used a homogeneous phantom in which the radiomics features of dose distribution were calculated, the important features identified in our study may be more robust against a variation of dose calculation parameters such as the dose calculation algorithm type, version, and dose calculation grid size.^34^, ^35^, ^36^, ^37^ Our present results demonstrated that the radiomic features for which a wavelet‐filter was applied were certainly necessary to optimize the performance of the machine learning models, since these dominated in the lists in Tables 7, 8, 9. The results also suggested that the bin size of each radiomic feature must be carefully selected in order to optimize the performance of the models. This point was not discussed in detail in previous studies.

Our study included the five categories of treatment sites as presented in Table 1 which are associated with various level of complexity of VMAT plan. For example, head‐and‐neck or whole pelvis plans are likely to be rather complex than prostate plans. The created models can be considered more comprehensive and versatile about the plan complexity than a treatment site specific model. Moreover, the results of our study indicated that the radiomic features of dose distribution were confirmed to be an indicator of the plan complexity.

The improvement of VMAT plans by the use of models for predicting dose evaluation metrics may provide a clinical benefit in the sense that the dose uncertainty in VMAT delivery to patients can be minimized.^43^ Our present findings also indicate the feasibility of improving VMAT plans by applying a radiomic analysis of dose distributions to predict the results of patient‐specific QA in the treatment planning process. The approach we adopted in this study may have an important benefit in that the dose evaluation metrics can be accurately predicted by only the dose distribution and may be easily implemented on a TPS. For example, radiomics features of dose distributions are expected to be optimized in the VMAT optimization process so that the resulting dose distribution would provide a high passing rate of a dose evaluation metric of patient‐specific QA.

There are several study limitations to address. First, our results may not be adaptable for cases in which types of detector arrays other than the Delta4 are used. Several authors have indicated that the differences in the geometry of detectors may lead to differences in GPR values even for the same VMAT plan. For example, Li et al. found that the correlation with plan complexity metrics was higher for GPRs measured by a Delta4 than for GPRs measured using an ArcCHECK2 array.^44^ Steer et al. showed that the Delta4 had higher sensitivity for error detection than the ArcCHECK.^45^ These studies suggested that among the variety of detectors used in patient‐specific QA, the Delta4 detector has at least non‐inferiority in predicting GPRs and error detection sensitivity. Secondly, the performances of our developed models may have depended specifically on the patient cohort we used, meaning that the models may be site‐specific and the same approach could have led to the different levels of performance at other institutes. To overcome this problem, it may be useful to carry out a multi‐institutional study to improve the generalization capability of our developed machine learning models.

## 5 CONCLUSIONS

We developed machine learning models to predict the values of the gamma, dose‐difference, and distance‐to‐agreement passing rates of patient‐specific QA for VMAT based on the radiomic features of 3D dose distribution in a dummy phantom. The developed models showed good performance and were comparable to the findings of the previous studies overall. It is advantageous to focus on a high dose region for improve the prediction accuracy. For certain metric and criteria, creating a model applicable for patients’ heterogeneity by training only with dose distributions in the phantom was possible by focusing on a high dose region. Our results demonstrate that the radiomic features of dose distribution can be considered good indicators of the plan complexity and useful in predicting measured dose evaluation metrics. The results of our study can provide a useful method to reduce the dosimetric uncertainty of VMAT plans by evaluating only the dose distribution.

## AUTHOR CONTRIBUTIONS

All authors satisfy the authorship requirements and do not have anything to disclose.

## CONFLICT OF INTEREST STATEMENT

The authors declare no conflicts of interest.
